# Validating the Chinese version of the Apathy Motivation Index and network analysis of apathy subtypes in a healthy Chinese sample

**DOI:** 10.3758/s13428-025-02686-3

**Published:** 2025-05-12

**Authors:** Xingyu Zhao, Liping Cui, Yunlin Sun, Lei Wang, Ke Wu, Qiangyan Che, Junyu Mao, Liuzhenxiong Yu, Pingping Liu, Panpan Hu, Kai Wang, Fengqiong Yu, Rong Ye

**Affiliations:** 1 Department of Neurology, The First Affiliated Hospital of Anhui Medical University, Hefei, 230022 China; 2 School of Mental Health and Psychological Sciences, Anhui Medical University, Hefei, 230032 China; 3 Anhui Province Key Laboratory of Cognition and Neuropsychiatric Disorders, Hefei, 230022 China; 4 Collaborative Innovation Center of Neuropsychiatric Disorders and Mental Health, Hefei, 230032 China; 5 Institute of Artificial Intelligence, Hefei Comprehensive National Science Center, Hefei, 230088 China; 6 Anhui Provincial Institute of Translational Medicine, Hefei, 230032 China; 7 Research Center for Translational Medicine, The Second Hospital of Anhui Medical University, Hefei, 230601 China; 8 Department of Neurology, The Second Affiliated Hospital of Anhui Medical University, Hefei, 230601 China

**Keywords:** Apathy, Motivation, Chinese translation, Motivational disorders, Network analysis

## Abstract

Apathy is a neuropsychiatric syndrome associated with various negative outcomes in patients with motivational disorders, commonly examined in multiple dimensions including cognitive, behavioral, and emotional domains. While there are some tools to measure apathy in China, there is a lack of appropriate instruments specifically designed to assess social motivation for a more comprehensive definition of apathy. Moreover, there is limited research on the complex interrelations among the intrinsic domains of apathy. Therefore, we developed the Chinese version of the Apathy Motivation Index (AMI) and assessed its reliability and validity in a sample of 758 participants. Network analysis was employed to explore the relationships among different domains of apathy. The Chinese version of the AMI classifies apathy into three domains, namely, behavioral activation, social motivation, and emotional sensitivity, which is consistent with the original scale; it also has good reliability and validity, making it suitable for measuring apathy and motivation in both healthy and patient populations. Additionally, network analysis revealed that apathy within the behavioral activation domain critically contributes to the overall measurement of apathy, while the social motivation dimension may serve as a bridge connecting other dimensions. Our study offers a reliable instrument to investigate apathy in Chinese-speaking individuals and could provide new insights for a better understanding of apathy in neuropsychiatric disorders.

**Supplementary Information:**

The online version contains supplementary material available at 10.3758/s13428-025-02686-3.

## Introduction

Apathy is a neuropsychiatric symptom typically defined as a decrease in or absence of motivation, characterized by a reduction in self-initiated goal-directed activities (Chong, 2020). Apathy is prevalent in most neurocognitive, neurodegenerative, and psychiatric disorders, typically associated with accelerated cognitive decline, impaired daily functioning, reduced quality of life, and consequently poor prognosis (Dauphinot et al., 2015; Miller et al., 2021; Robert et al., 2018). Apathy can also be widely observed in healthy populations (Ang et al., 2017; Bonnelle et al., 2015; Brodaty et al., 2010), indicating that targeted tools for measuring apathy are needed to effectively capture the motivational deficits in both clinical and healthy populations.

The high comorbidity rates and negative impacts of apathy place significant pressure on public health and socioeconomic systems, a problem especially pronounced in China due to its large population. The comorbidity rates of apathy are significantly high in Parkinson’s disease (PD) (D’Iorio et al., 2018), Alzheimer’s disease (AD) (Robert et al., 2010), schizophrenia, and depression, reaching levels of 40% to 70% (Le Heron et al., 2019). Taking AD as an example, according to data from the Global Burden of Disease (GBD) study, the number of people worldwide living with AD and related dementias reached 51.62 million in 2019. In the same year, the total number of individuals with AD and related dementias in China was 13.14 million, accounting for approximately 25.5% of the global AD patient population (National Center for Neurological Disorders et al., 2024). Research indicates that 61.4% of AD patients in China exhibit apathy (Zhao et al., 2012), suggesting that millions of individuals affected by apathy are among those with AD and related dementias. Therefore, accurately identifying and effectively intervening in cases of apathy is crucial for improving the quality of life in patients with neuropsychiatric symptoms, slowing cognitive decline, and improving the prognosis of AD and other neurodegenerative disorders in China.

The diagnostic definition of apathy has evolved over the past two decades. Apathy was originally characterized into three subdomains: apathy in goal-directed behavior, apathy in goal-directed cognitive activities, and emotional apathy (Robert et al., 2009). In 2018, the diagnostic criteria for apathy were revised in the annual meeting of the European Psychiatric Association, which consolidated goal-directed behavior and cognitive activities into a single domain and introduced a new domain for social interaction according to the latest research (Robert et al., 2018). Although many researchers have extensively studied the different dimensions of apathy, very few studies have explored the relationships between these dimensions. Apathy manifests with symptoms across behavioral, cognitive, emotional, and social domains, and the interactions among these symptoms may be more complex (Robert et al., 2009, 2018). Network analysis, which is grounded in psychopathological theoretical concepts, treats psychological and cognitive characteristics as nodes and the relationships between different nodes as edges, thereby allowing for the identification of core symptoms and the relationships between them (Levinson et al., 2018). The advantage of network analysis lies in its graphical visualization, allowing for a more intuitive description of the relationships between causal variables through graph theory. By viewing apathy as a multidimensional syndrome and considering its various dimensions as nodes, constructing a network of apathy symptoms may provide new insights into understanding apathy.

Currently, several measurement tools are utilized in China to evaluate apathy, including the Apathy Evaluation Scale (Marin et al., 1991), Dimensional Apathy Scale (Radakovic & Abrahams, 2014), Lille Apathy Rating Scale (Sockeel et al., 2006), and Neuropsychiatric Inventory-Apathy subscale (Cummings et al., 1994), which do not specifically address measurements of social motivation. A recent development of the Apathy Motivation Index (AMI) divided apathy into three domains: behavioral activation, emotional sensitivity, and social motivation. This scale conforms to the latest criteria for diagnosing apathy and has good reliability and validity (Ang et al., 2017). In addition, there is a child version of the scale (Hewitt et al., 2024), which can be combined with the adult version to measure the cycle of apathy throughout life, and the scale has been translated into multiple languages (Altieri et al., 2023; Corveleyn et al., 2023).

The purpose of the current study is to translate and validate the AMI to fill the gap in apathy measurement in China and to provide diagnostic value for different domains of apathy. In addition, network analysis techniques were used to construct the apathy network and identify its core nodes and potential relationships in Chinese populations.

## Methods

### Participants

This study recruited participants via offline and online sources. To ensure data quality, we included four attention check questions in all questionnaires (e.g., “Please select the third option for this question”). Additionally, during online recruitment, we controlled for factors such as participants’ credit scores, IP addresses, and geographical locations. Participants who successfully completed the online questionnaire received compensation of 15.5 yuan (approximately 2 USD). A total of 909 participants were recruited, 151 of which were excluded due to refusal to answer or failure to pass the attention check questions. Ultimately, 758 participants (303 male, 455 female; mean age = 27.18 years, range 18–63) completed the Chinese version of the AMI. Among them, 359 participants were recruited offline (from Anhui Medical University), while 399 were sourced online from the Chinese Credamo platform data marketplace (www.credamo.com).

To examine the factor structure of the Chinese version of the AMI, we randomly divided the 758 participants into two samples: sample 1, consisting of 379 individuals (148 male, 231 female; mean age = 26.90 years, range 18–62), and sample 2, consisting of 379 individuals (155 male, 224 female; mean age = 27.47 years, range 18–63). There were no significant differences between the two samples in terms of AMI scores (*p* = 0.672 > 0.05), gender (*p* = 0.604 > 0.05), or age (*p* = 0.421 > 0.05). Furthermore, to assess the test–retest reliability of the Chinese version of the AMI, 58 participants were additionally recruited at Anhui Medical University. A total of 47 participants successfully passed the attention check questions and completed the Chinese version of the AMI on two occasions, with a 6–8-day interval between the assessments. All participants were native Chinese speakers, and each provided informed consent approved by the Biomedical Ethics Committee of Anhui Medical University (Ethics Approval number 83220373, Approval date: May 30, 2022).

### Chinese version of the AMI

This study followed the cross-cultural translation guidelines for self-report measures (Beaton et al., 2000). After permission was obtained from the original authors of the AMI, the questionnaire was translated from English to Chinese and then back-translated via the following steps: (1) Two experts with psychology degrees who were proficient in English translated the original English version of the AMI scale; this was followed by a back-translation conducted by two English majors who had no previous experience with the scale. The four translators compared the two translation versions with the original scale, discussed their differences, and reached a consensus in developing the initial Chinese version of the AMI. (2) A pilot survey was conducted offline at Anhui Medical University using 10 copies of the initial Chinese version of the AMI to collect feedback and suggestions for modifications, ensuring the absence of semantic ambiguities or other language-related issues. (3) An expert panel of two psychologists with doctoral degrees evaluated and refined the initial version of the AMI, ultimately producing the final Chinese version of the AMI.

To validate the effectiveness of the Chinese version of the AMI, we collected 53 valid questionnaires at Anhui Medical University. We found that the scale’s overall reliability was satisfactory (Cronbach’s α = 0.730). After validating the effectiveness of the Chinese version of the AMI, we formally administered it. All participants in this pilot study signed informed consent forms and received approval from the ethics committee (Ethics Approval number 83220373, Approval date: May 30, 2022).

### Materials and procedure

The following is a set of scales that participants were required to complete.

- Apathy Motivation Index (AMI) (Ang et al., 2017): The original English version of the AMI comprises 18 items. Apathy is divided into three subscales: behavioral activation (items 5, 9, 10, 11, 12, and 15), social motivation (items 2, 3, 4, 8, 14, and 17), and emotional sensitivity (items 1, 6, 7, 13, 16, and 18). The scale employs a five-point Likert scale (0–4), where higher scores indicate higher levels of apathy. The level of apathy is assessed by averaging the ratings of the items on the subscale. (The participants completed the Chinese version of the AMI, which also consists of 18 items).
- Apathy Evaluation Scale-Self (AES-S) (Marin et al., 1991): The scale comprises 18 items, each rated on a 1–4-point scale. Items 6, 10, and 11 are reverse-scored. The total score is calculated by summing the scores of all the items, with higher scores indicating a greater degree of apathy.
- Beck Depression Inventory-II (BDI-II) (Beck et al., 1996): This scale consists of 21 items designed to assess the severity of depression. Each item is scored on a scale from 0 to 3, with the total score being the sum of all individual item scores. The higher the total score, the more severe the depression.
- Snaith-Hamilton Pleasure Scale (SHAPS) (Franken et al., 2007): The scale consists of 14 items, requiring participants to rate their agreement with various pleasant scenarios related to their feelings of joy. Each item is assessed on a four-point Likert scale: (1 = “strongly agree,” 2 = “agree,” 3 = “disagree,” 4 = “strongly disagree”). The total score is calculated by summing the scores of all the items, with higher total scores indicating a greater lack of pleasure.
- Generalized Anxiety Disorder (GAD) (Spitzer et al., 2006): This scale consists of seven items used to evaluate the frequency of anxiety symptoms over the past 2 weeks, with each item rated on a four-point scale. The total score is the sum of all item scores, with a higher total score indicating more severe anxiety.

### Statistical analysis

Data analysis was performed using IBM SPSS Statistics version 26.0, with confirmatory factor analysis conducted using Amos version 21.0. Network analysis was carried out via R software (version 4.2.1). To test the quality of the items, we conducted item‒total correlation analyses on the translated AMI scale items. We interpreted effect sizes based on Cohen’s conventions (weak, *r* < 0.30; moderate, *r* = 0.30–0.50; strong, *r* > 0.50) (Cohen, 1988). Items with low item‒total correlations (*r* < 0.30) were excluded from further analysis. We employed a two-stage factor analysis approach to assess the structural validity of the Chinese version of the AMI. We used the Kaiser‒Meyer‒Olkin (KMO) test (Kaiser, 1974) and Bartlett’s test of sphericity to ensure that the AMI items had sufficient intercorrelations for appropriate factor analysis. In the exploratory factor analysis (EFA) using sample 1, the EFA was conducted with a principal component analysis and varimax rotation. We selected items with loadings greater than 0.40 and tested items with loadings between 0.30 and 0.40 (Corveleyn et al., 2023).

For the confirmatory factor analysis (CFA) using sample 2, we analyzed the data to verify the factor structure identified in the EFA. We assessed model fit via three indices: the root mean square error of approximation (RMSEA), the standardized root mean square residual (SRMR), and the comparative fit index (CFI). In general, lower values of RMSEA and SRMR indicate better model fit, whereas higher values of the CFI typically suggest a better fit (Cangur & Ercan, 2015). We used traditional criteria for model fit assessment to evaluate the Chinese version of the AMI, specifically requiring that RMSEA and SRMR be less than 0.08 and CFI be greater than 0.90 (Xia & Yang, 2019). Given that the participants were a healthy population with a skewed distribution of responses, we employed the MLR (maximum likelihood estimation in robust statistics) method to calculate the RMSEA, SRMR, and CFI in order to avoid potential bias. For the external validity of the AMI, we used Pearson correlation coefficients as indicators to assess the relationship. We calculated the Pearson correlation coefficients between the total score and subscales of the AMI and the total scores of the AES, BDI, SHAPS, and GAD. To evaluate the internal consistency reliability of the assessment of the AMI, we calculated Cronbach’s alpha and Guttman’s half-score coefficients for both the total score and the subscales. Additionally, we assessed the test–retest reliability by computing the intraclass correlation coefficient (ICC) values between the AMI total score and the subscales. For internal consistency reliability, we considered Cronbach’s alpha and Guttman’s split-half coefficients above 0.60 to indicate acceptable reliability (Bullinger et al., 1996). Regarding test–retest reliability, an ICC value greater than 0.7 was deemed necessary (Shrout & Fleiss, 1979).

For the network analysis of the AMI, we employed a regularized partial correlation network. We estimated the cross-sectional network of symptoms via the Gaussian graphical model (GGM) and applied the graphical least absolute shrinkage and selection operator (GLASSO) for regularization to reduce false-positive edges. Following the recommendations of Epskamp and Fried (2018), we set the tuning parameter to 0.5 to balance sensitivity and specificity and utilized the R package *qgraph* for network visualization (Epskamp et al., 2012). The relationships between nodes are represented by the color of the edges (with green indicating positive relationships and red indicating negative relationships), whereas the strength of the relationships (i.e., edge weight) is indicated by the thickness of the edges. We employed the *networktools* package in R to calculate network centrality metrics, selecting the “strength” metric to assess the importance of nodes within the network. A higher strength value indicates that the node is more central to the network. (Robinaugh et al., 2016). In addition, we used exploratory graph analysis (EGA) to identify the communities within the Chinese version of the AMI, which also served as a reexamination of the dimensional structure of the AMI. We conducted the EGA analysis using the EGA package in R, selecting the *glasso* model (GLASSO with extended Bayesian information criterion [EBIC]) for model fitting. For community detection, we employed the Louvain method. After defining the communities within the network, we analyzed bridging symptoms by calculating the bridge strength. Nodes with higher bridge strength values are more strongly associated with other communities. To assess the stability of the community structure, we conducted a stability test for the items using the *bootEGA* package in R, performing 2,500 iterations in total (Christensen & Golino, 2021). To assess the stability of the network, we employed the *bootnet* package to conduct a nonparametric bootstrap test (*N* = 2,500) to calculate the 95% confidence intervals (CIs) for the edge weights within the network, thereby demonstrating the accuracy of the edge weight estimates. A narrower confidence interval indicates a more accurate estimation of edge weights, enhancing the accuracy of centrality measures. We used the *corStability* function to calculate the centrality stability (CS) coefficient of the network. A CS coefficient value greater than 0.5 indicates good stability of centrality, with an acceptable minimum value set at 0.25 (Epskamp et al., 2018).

### Statistical power and sample size determination

The sample size in this study (*N* = 758) was sufficient for EFA based on recent recommendations (Mundfrom et al., 2005). In the initial research, the ratio of variables to factors for AMI was 6:1. Assuming this ratio remains constant, and in accordance with the recommendations of Mundform et al. (2005), a minimum of 160 participants is required (assuming a three-factor solution) to ensure excellent replicability. The number of participants in this study far exceeds the minimum sample size requirement, thus enabling high levels of replicability to be achieved.

## Results

### Participant characteristics

In this study, 758 participants completed the Chinese version of the AMI and other scales. The participants’ demographic information and scores on the scales are shown in Table 1.

**Table 1:** Demographics and questionnaire scores for study samples

	Sample 1 (*N* = 379)	Sample 2 (*N* = 379)	Total sample (*N* = 758)
Gender, M/F	148/231	155/224	303/455
Age, years	26.90(9.25)	27.47(10.11)	27.18(9.69)
Education, years	16.19(1.22)	16.23(1.01)	16.21(1.12)
AES (14–72)	30.91(7.69)	30.18(8.41)	31.05(8.06)
BDI (0–63)	8.49(10.21)	8.76(10.20)	8.62(10.20)
GAD (0–21)	3.75(4.16)	3.78(4.18)	3.77(4.17)
SHAPS (14–56)	23.8(5.70)	24.08(6.16)	23.96(5.93)
AMI (0–4)	1.19(0.52)	1.15(0.54)	1.17(0.53)
AMI-BA (0–4)	1.10(0.65)	1.13(0.73)	1.11(0.69)
AMI-SM (0–4)	1.50(0.69)	1.46(0.73)	1.48(0.71)
AMI-ES (0–4)	0.98(0.59)	0.86(0.56)	0.92(0.58)

Scores on the scale are presented as mean (SD). *AES *Apathy Evaluation Scale-Self, *BDI *Beck Depression Inventory-II, *GAD *Generalized Anxiety Disorder, *SHAPS *Snaith-Hamilton Pleasure Scale, *AMI *Apathy Motivation Index, *BA *behavioral activation, *ES *emotional sensitivity, *SM *social motivation

### Item analysis

An item‒total correlation analysis was conducted on the 18 items of the Chinese version of the AMI for the 758 participants, and the results are presented in Table 2. The absolute correlations between each item and the total score were generally moderate to high (*r* = 0.418–0.691) except for the first and the sixth items. Specifically, the first item had a relatively low correlation with the total score (*r* = 0.102). After corrected item–total correlation, it was found to be unrelated (*r* = − 0.01), and this item was excluded from further analysis. Although the sixth item showed a negative correlation with the total score (*r* = − 0.249), its correlation strength reached a moderate level after adjustment (*r* = − 0.369); therefore, it was temporally retained for further analysis.

**Table 2:** Apathy Motivation Index (AMI) item characteristics

Item	Item–total correlation	Corrected item–total correlation	Cronbach’s alpha if item removed
1. I feel sad or upset when I hear bad news	0.102^**^	**− 0.01**	**0.828**
2. I start conversations with random people	0.584^**^	0.482^**^	0.801
3. I enjoy doing things with people I have just met	0.574^**^	0.476^**^	0.801
4. I suggest activities for me and my friends to do	0.658^**^	0.586^**^	0.795
5. I make decisions firmly and without hesitation	0.617^**^	0.531^**^	0.797
6. After making a decision, I wonder if I have made the wrong choice	− 0.249^**^	− 0.369^**^	**0.855**
7. Based on the last two weeks, I would say I care deeply about what my loved ones think of me	0.418^**^	0.317^**^	0.811
8. I go out with friends on a weekly basis	0.554^**^	0.441^**^	0.804
9. When I decide to do something, I am able to make an effort easily	0.656^**^	0.587^**^	0.795
10. I do not like to laze around	0.602^**^	0.523^**^	0.799
11. I get things done when they need to be done, without requiring reminders from others	0.634^**^	0.565^**^	0.797
12. When I decide to do something, I am motivated to see it through to the end	0.677^**^	0.622^**^	0.796
13. I feel awful if I say something insensitive	0.432^**^	0.354^**^	0.809
14. I start conversations without being prompted	0.595^**^	0.524^**^	0.800
15. When I have something I need to do, I do it straightaway so it is out of the way	0.691^**^	0.626^**^	0.793
16. I feel bad when I hear an acquaintance has an accident or illness	0.481^**^	0.411^**^	0.806
17. I enjoy choosing what to do from a range of activities	0.591^**^	0.519^**^	0.800
18. If I realize I have been unpleasant to someone, I feel terribly guilty afterward	0.461^**^	0.379^**^	0.807

^*^p < 0.05, **p < 0.01. Bold text indicates outlier items

### EPA and CFA

Exploratory factor analysis was conducted on the 17-item Chinese version of the AMI using sample 1 (*N* = 379). The Kaiser‒Meyer‒Olkin (KMO) value for sampling adequacy was 0.886, and the chi-square value of Bartlett’s test of sphericity was 2013.707 (*p* < 0.001), These results indicate that the data are suitable for factor analysis, allowing for the effective extraction of underlying factors. Principal component analysis was carried out on the 17 items with varimax rotation, extracting three factors labeled behavioral activation (BA), social motivation (SM), and emotional sensitivity (ES), which accounted for 50.792% of the variance. This is largely consistent with the three-dimensional model proposed in the original scale, with all items except the sixth item loading onto the intended dimensions (for specific loadings, see Table 3).

**Table 3:** Factor loadings of the 17 items of the Apathy Motivation Index

Original question number	Original dimensions	Factor 1	Factor 2	Factor 3
AMI- 11	Behavioral activation	**0.745**	0.099	0.182
AMI- 12	Behavioral activation	**0.705**	0.255	0.148
AMI- 15	Behavioral activation	**0.683**	0.266	0.179
AMI- 9	Behavioral activation	**0.660**	0.265	0.090
AMI- 10	Behavioral activation	**0.624**	0.024	0.337
AMI- 5	Behavioral activation	**0.601**	0.493	0.003
AMI- 6	Behavioral activation	**–0.516**	–0.239	0.193
AMI- 2	Social motivation	0.082	**0.737**	0.091
AMI- 3	Social motivation	0.228	**0.680**	0.028
AMI- 8	Social motivation	0.187	**0.668**	0.075
AMI- 4	Social motivation	0.324	**0.559**	0.281
AMI- 17	Social motivation	0.216	**0.519**	0.251
AMI- 14	Social motivation	0.362	**0.517**	0.068
AMI- 13	Emotional sensitivity	0.153	–0.029	**0.770**
AMI- 18	Emotional sensitivity	0.107	0.123	**0.725**
AMI- 16	Emotional sensitivity	0.229	0.146	**0.666**
AMI- 7	Emotional sensitivity	––0.101	0.332	**0.505**

Bold indicates factor loadings with absolute values greater than 0.5

The preliminary analysis revealed that the sixth item (“After making a decision, I will wonder if I have made the wrong choice,” emotional dimension) had a high negative factor loading in the behavioral dimension, indicating that the sixth item is conceptually opposite to the behavioral dimension. This does not align with the original scale’s definition of it being an emotional dimension. The factor loading of this item on the emotional dimension is low (0.193), with a weak item–total correlation (*r* = − 0.249). An attempt was also made to conduct confirmatory factor analysis (CFA); however, the model fit indices were not ideal. Based on these findings, the sixth item was excluded from further analysis. CFA was subsequently conducted on the 16-item Chinese version of the AMI using sample 2 (*N* = 379). The results revealed a χ^2^/*df* ratio of 3.169, with a CFI of 0.907. The SRMR and RMSEA values were 0.062 and 0.066, respectively, both of which are less than 0.08, indicating a good model fit.

### External validity

Table 4 summarizes the correlations between the total score and each dimension score of the Chinese version of the AMI with other scales, with correlation coefficients ranging from 0.16 to 0.765. The Chinese version of the AMI showed a strong positive correlation (*r* = 0.765) with the existing Chinese version of AES, indicating good convergent validity; it also correlated positively with BDI (*r* = 0.481), SHAPS (*r* = 0.642), and GAD (*r* = 0.373), demonstrating good discriminant validity. Overall, these findings suggest that the AMI has good external validity.

**Table 4:** Pearson correlation coefficients between AMI and criterion-related scales

	AMI	BA	SM	ES
AES	0.765^**^	0.747^**^	0.633^**^	0.415^**^
BDI	0.481^**^	0.461^**^	0.438^**^	0.222^**^
SHAPS	0.642^**^	0.554^**^	0.554^**^	0.407^**^
GAD	0.373^**^	0.385^**^	0.324^**^	0.160^**^

^*^p < 0.05, **p < 0.01 AES: Apathy Evaluation Scale-Self, BDI: Beck Depression Inventory-II, SHAPS: Snaith-Hamilton Pleasure Scale, GAD: Generalized Anxiety Disorder, AMI: Apathy Motivation Index, BA: behavioral activation, ES: emotional sensitivity, SM: social motivation

### Reliability

By combining sample 1 and 2 (*N* = 758), Cronbach’s alpha and Guttman’s split-half coefficients were calculated for the total score and subscales of the Chinese version of the AMI (16 items). The results showed that the AMI demonstrated good internal consistency reliability (Cronbach’s alpha: α_overall_ = 0.870, α_BA_ = 0.850, α_SM_ = 0.776, α_ES_ = 0.667) and split-half reliability (Guttman’s split-half coefficient: overall = 0.836, BA = 0.857, SM = 0.769, ES = 0.701). Furthermore, to avoid reliability inflation caused by sample size, we conducted reliability analyses again in two smaller samples, sample 1 (*N* = 379) and sample 2 (*N* = 379), with the following results: for sample 1, Cronbach’s alpha α_overall_ = 0.863, α_BA_ = 0.830, α_SM_ = 0.766, α_ES_ = 0.652 and split-half reliability (Guttman’s split-half coefficient: overall = 0.835, BA = 0.842, SM = 0.761, ES = 0.692); for sample 2, Cronbach’s alpha α_overall_ = 0.877, α_BA_ = 0.868, α_SM_ = 0.786, and α_ES_ = 0.667, and split-half reliability (Guttman’s split-half coefficient: overall = 0.836, BA = 0.870, SM = 0.780, ES = 0.706). The results indicated that the item dimensions we identified remained valid in both datasets, demonstrating that even in smaller samples, the Chinese version of the AMI still exhibits good reliability. After a 6–8-day interval, 47 participants completed the AMI at two time points. We calculated the ICCs for the total score of the AMI and its subscales. The test–retest reliability of the Chinese version of the AMI also showed good performance, with ICC_overall_ = 0.824 (0.685–0.902), ICC_BA_ = 0.874 (0.774-–0.930), ICC_SM_ = 0.815 (0.668–0.897), and ICC_ES_ = 0.834 (0.702–0.908).

### Cutoff thresholds

We provided reference values for diagnosing indifference on the total score and dimensions of the Chinese version of the AMI. Indifference is considered moderate if it exceeds one standard deviation and severe if it exceeds two standard deviations, as shown in Table 5.

**Table 5:** Proposed cutoffs for moderate (> 1 SD) and severe (> 2 SD) apathy on AMI

AMI subscale	Mean ± SD	+ 1 SD	+ 2 SD
Total	1.17 ± 0.53	1.70	2.23
Behavioral activation	1.11 ± 0.69	1.80	2.49
Social motivation	1.48 ± 0.71	2.19	2.90
Emotional sensitivity	0.92 ± 0.58	1.5	2.08

The total score of the AMI and the subscale scores are expressed as mean scores, ranging from 0 to 4, with higher scores indicating greater apathy. The study included 758 participants (mean age: 27.18 ± 9.69 years), with the majority being approximately 20 years old. The cutoff values presented are for reference purposes only

### Network analysis of AMI

**Fig. 1:**
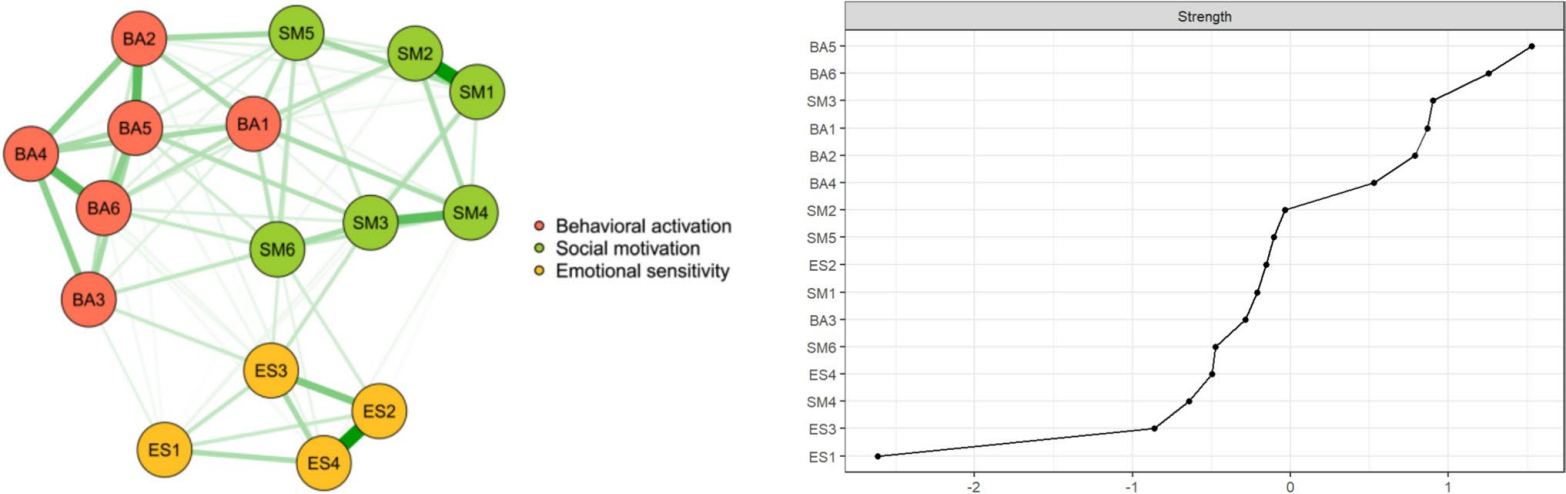
Apathy Motivation Index regularized network graph and strength value graph (*n* = 758). Left panel shows the apathy network, which illustrates the relationships among behavioral activation, social motivation, and emotional sensitivity. The strength values in the right panel are sorted in numerical order after standardization. BA5 has the highest strength value and is the key connecting node of the network. The behavioral activation dimension is represented by BA1–6, corresponding to original items 5, 9, 10, 11, 12, and 15. The social motivation dimension is indicated by SM1–6, linked to items 2, 3, 4, 8, 14, and 17, while emotional sensitivity is represented by SM1–4, corresponding to items 7, 13, 16, and 18

The standardized strength values of the regularized partial correlation network and network nodes are shown in Fig. 1. In this network, there are 16 nodes and 77 nonzero edges. The node with the highest strength value is BA5 (“When I decide to do something, I am motivated to see it through to the end.”), with strength of 1.07, followed by BA6 (“When I have something I need to do, I do it straightaway so it is out of the way”), with strength of 1.03.

**Fig. 2:**
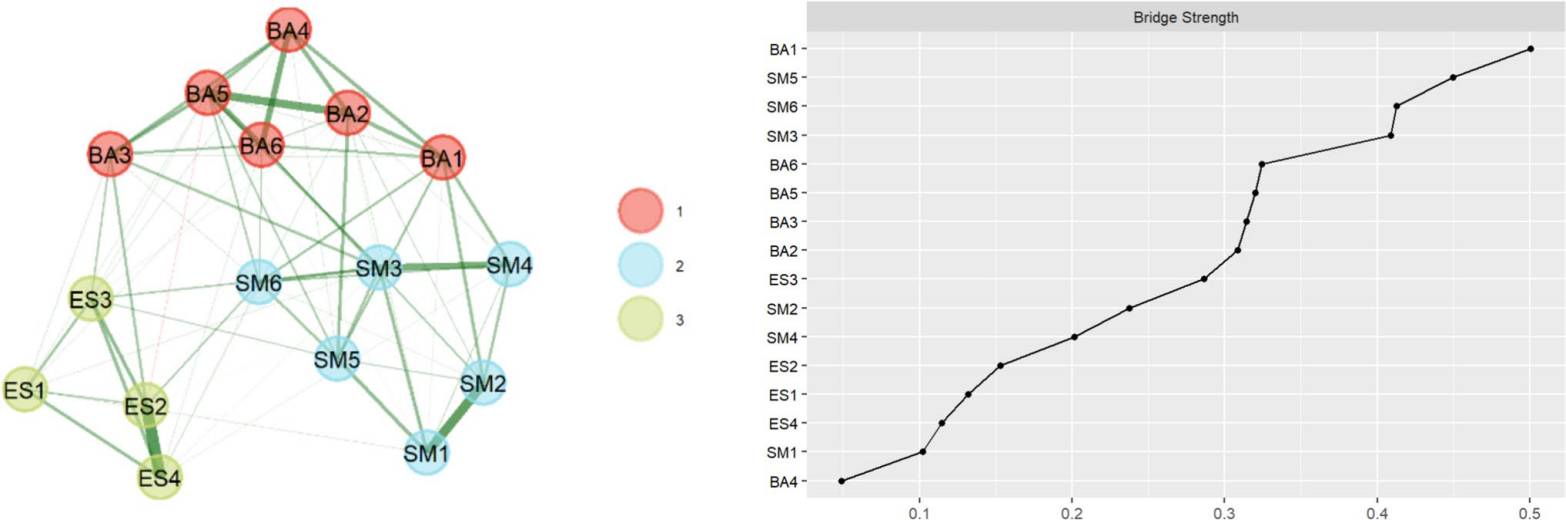
The exploratory graph analysis (EGA) and corresponding standardized bridge strength values of network nodes (*n* = 758). On the left is the result graph of the EGA, where 1, 2, and 3 represent different dimensions. The results are consistent with the factor analysis results. On the right is the bridge strength values chart, where the calculated bridge strength values are sorted in descending order. In the network’s bridge nodes, BA1 has the highest expected influence values, followed by SM5. BA1–6 correspond to the original items 5, 9, 10, 11, 12, and 15; SM1–6 correspond to the original items 2, 3, 4, 8, 14, and 17; ES1–4 correspond to the original items 7, 13, 16, and 18

The results of the exploratory graph analysis (EGA) and the standardized bridge strength values of the network nodes are presented in Fig. 2. The EGA results indicate that the Chinese version of the AMI should be divided into three factors, and this dimensional structure is consistent with the findings of previous factor analyses. The highest bridge strength value is BA1 (“I make decisions firmly and without hesitation”), followed by SM5 (“I start conversations without being prompted”). This indicates that they are more important as bridging nodes within the apathy network.

**Fig. 3:**
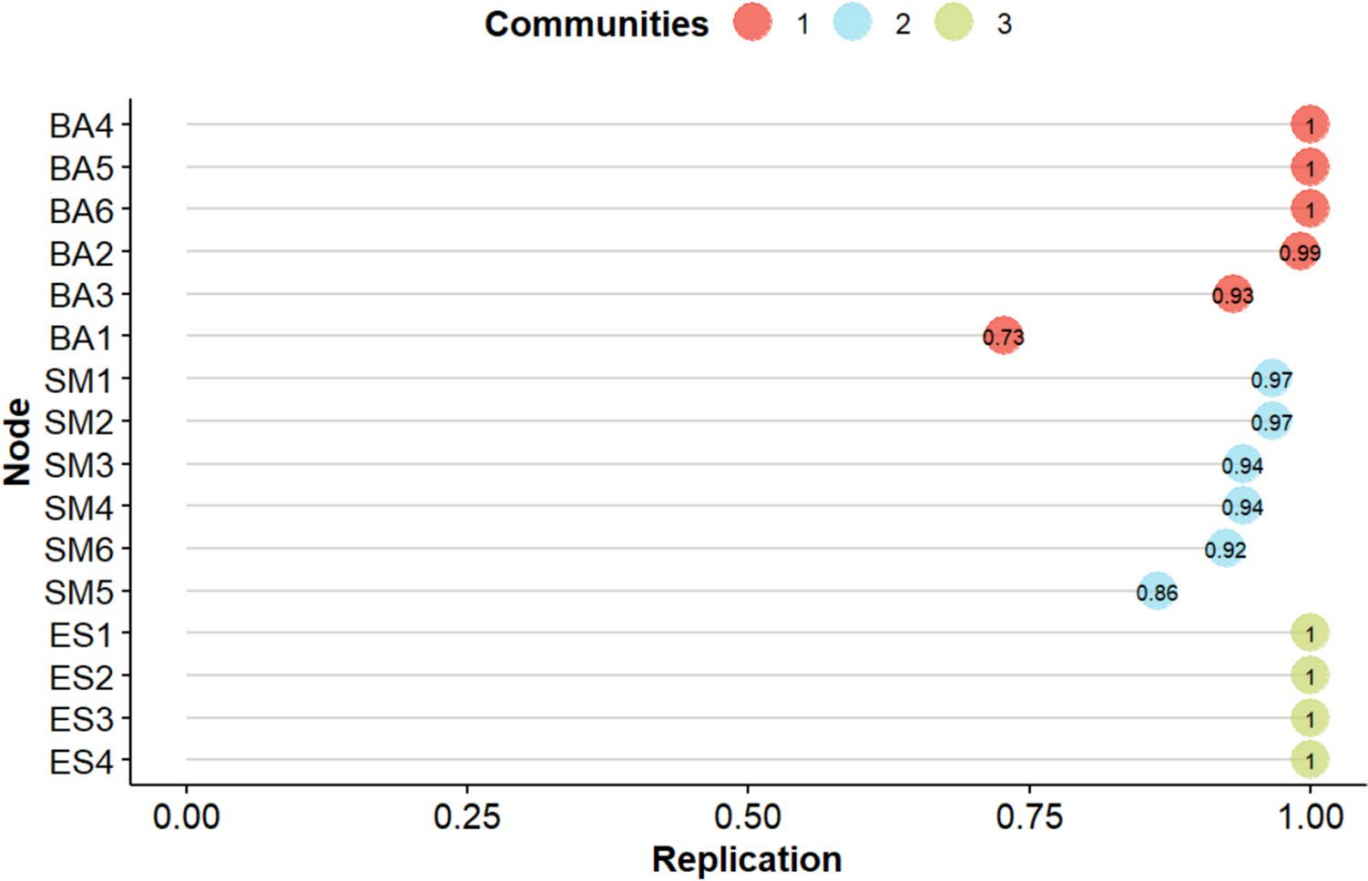
Item stability of the Apathy Motivation Index. The horizontal axis represents the project stability results after 2,500 iterations, with larger values indicating greater project stability. The vertical axis represents different items of AMI. Community 1, 2, and 3 represent different dimensions of the AMI. BA1–6 correspond to the original items 5, 9, 10, 11, 12, and 15; SM1–6 correspond to the original items 2, 3, 4, 8, 14, and 17; ES1–4 correspond to the original items 7, 13, 16, and 18

The stability test results for the EGA are shown in Fig. 3. The results indicate that all items of the Chinese version of the AMI loaded stably onto their respective hypothesized dimensions (all item loadings > 0.70) (Rader et al., 2023), confirming the good stability of the dimensions identified by the EGA analysis.

The accuracy test of the network reveals that the confidence intervals for edge weights at a 95% confidence interval are relatively narrow, indicating a precise assessment of edge weights (please refer to the appendix). The stability test of the network shows a CS coefficient of 0.749 for strength and a CS coefficient of 0.673 for bridge strength, both exceeding 0.5, indicating that these two indicators have a high level of stability (please refer to the appendix).

## Discussion

The purpose of this study was to translate the original English AMI into Chinese in order to provide a cross-cultural, cross-ethnic, and cross-regional tool for assessing levels of apathy in healthy individuals and patients among Chinese-speaking populations. Additionally, the study aimed to utilize network analysis methods to explore the core nodes of apathy. The Chinese version of the AMI has good reliability and validity, making it suitable as a formal assessment tool for assessing apathy levels.

Consistent with previous research, the Chinese version of the AMI revealed three common factors: behavioral activation, social motivation, and emotional sensitivity. All the items were loaded onto their respective hypothesized dimensions, and confirmatory factor analysis resulted in a well-fitting model, indicating that the Chinese version of the AMI effectively distinguishes between different domains of apathy. The original AMI consisted of 18 items, 16 of which were retained in the final version. The first item of the original scale (“I feel sad or upset when I hear bad news,” emotional dimension) was excluded due to low overall item correlation. This may be attributed to cultural differences. Eastern cultures tend to emphasize collectivism, while Western cultures are more inclined towards individualism. When confronted with negative news, individuals in Western contexts may focus more on personally relevant information, whereas those in Eastern contexts may also consider negative events that are less directly related to themselves. In the context of Chinese culture, bad news can be understood differently based on its relevance to the individual. The first type involves negative news directly impacting the person, such as failing an exam or a serious illness. The second type pertains to bad news related to those in the individual’s social circle, such as accidents experienced by friends or acquaintances, which is similar to the 16 th item of the original scale (“I feel bad when I hear an acquaintance has an accident or illness,” emotional dimension). The third type encompasses bad news that has little personal relevance, such as an airline crash in a distant location or forest fires. This creates a sense of uncertainty for the participants. Therefore, participants may make varied choices based on their understanding of the bad news presented to them. Notably, we also found that the option distribution for the first item of the original scale was more dispersed than that for the 16 th item of the original scale (the second type of bad news). During the offline survey process, some participants also expressed confusion by asking, “What kind of bad news is it?” when responding to the first item. In summary, we have excluded the first item from the final version.

The sixth item of the original scale (“After making a decision, I will wonder if I have made the wrong choice,” emotional dimension) was excluded from the Chinese version of the AMI due to several concerns, as follows: (1) This item exhibits high negative loading in the behavioral activation dimension and low positive loading in the emotional sensitivity dimension in both our Chinese and the French version of the AMI (Corveleyn et al., 2023). (2) In the Italian version of the AMI (Altieri et al., 2023), the sixth item had no significant overall item correlation. (3) In the initial study (Ang et al., 2017), the sixth item had the lowest factor loading among all items. (4) From a semantic perspective, reevaluation after making a decision may better reflect a person’s decisiveness in behavior rather than emotional experience. Therefore, this item may be difficult to consolidate within any domains of apathy, and we decided to exclude it from the final Chinese version of the AMI.

Regarding the convergent validity of the Chinese version of the AMI, as expected, there was a high correlation between the AMI score and the AES score, indicating that the AMI can effectively measure apathy. Additionally, the AMI showed a moderate correlation with the GAD and the BDI, suggesting that apathy may be related to anxiety and depression, which is consistent with the findings of most studies (Altieri et al., 2023; Ang et al., 2017; Le Heron et al., 2019). Notably, we observed a strong correlation between apathy and anhedonia, a phenomenon reported in other studies (Gunaydin et al., 2014). Apathy is often conceptualized as a loss of motivation, whereas anhedonia refers to a persistent and significant reduction in interest or pleasure in nearly all daily activities, including the motivational component characterized by a loss of interest in taking action to seek pleasure (Chan et al., 2012). Recent research suggests that there might be some common mechanisms underlying both apathy and anhedonia (Husain & Roiser, 2018). Like apathy, anhedonia can also be divided into various dimensions, such as social interactions, food enjoyment, consummatory experiences, and anticipatory pleasure (Chan et al., 2012; Franken et al., 2007). By distinguishing between apathy and anhedonia in different domains, it may be possible to explore the similarities of these syndromes at a more nuanced level. The AMI is consistent with the latest diagnostic definitions of apathy and can serve as an effective measurement tool for apathy. Combined with other anhedonia assessment tools, it may facilitate the exploration of the shared neurobiological mechanisms underlying apathy and anhedonia.

In the regularized partial correlation network of the AMI, the items within each dimension are closely interconnected. BA5 (“When I decide to do something, I am motivated to see it through to the end”) and BA6 (“When I have something I need to do, I do it straightaway so it is out of the way”) showed the highest importance within all nodes of the apathy network, with the highest strength values. Both items belong to the behavioral activation dimension, and other items in this dimension also present high strength values, indicating that the measurement of behavioral activation has the greatest impact on apathy. In terms of bridge strength, BA1 (“I make decisions firmly and without hesitation”) has the highest bridge strength value, followed closely by SM5 (“I start conversations without being prompted”), SM6 (“I enjoy choosing what to do from a range of activities”), and SM3 (“I suggest activities for me and my friends to do”). This suggests that decisiveness in decision-making and proactivity in communication strongly influence the overall apathy network. This finding indicates that social motivation is a crucial dimension that links the behavioral activation and emotional sensitivity dimensions. In the context of AMI, social motivation is defined as the level of engagement in social interactions. A high level of social interaction typically indicates a greater frequency of social behaviors and emotional needs, encompassing both behavioral activation and emotional sensitivity. Social motivation is closely related to quality of life and often plays a crucial role in the workplace, influencing individuals’ choices between competitive and cooperative behaviors. High-quality social interactions are also a key factor in enhancing life satisfaction (Grant & Shandell, 2022). Therefore, in the apathy field, social motivation may be a potential therapeutic perspective.

This study has several limitations. The sample primarily consists of young individuals with a relatively high level of education, and thus the current findings could be less representative of the elderly population. Therefore, it is essential to include older participants and individuals from diverse patient groups in future research to enhance the comprehensiveness of the findings. Furthermore, the network analysis in this study relied solely on the AMI to construct the apathy network, limiting the generalizability of the conclusions. Future research could benefit from incorporating multiple measures of apathy to provide a more robust analysis, and combining other scales may help explore the potential common and distinctive mechanisms underlying various symptoms in motivational disorders.

In conclusion, the AMI is a reliable and valid tool for assessing apathy and motivation, providing a dependable means of measurement in the field of social motivation and apathy in Chinese-speaking individuals. It can be widely utilized in various contexts, including apathy screening in elderly community settings, psychological assessments for staff, and public health surveys. The network analysis based on the AMI indicates that behavioral activation is the most effective indicator of apathy severity. In contrast, social motivation has the most substantial impact on overall levels of apathy; this suggests that we may consider apathy as a syndrome from the perspective of social motivation.

## Supplementary Information

Below is the link to the electronic supplementary material.

Supplementary file1 (DOCX 757 KB)

## Data Availability

The dataset and code supporting this research conclusion can be accessed at the Science Data Bank via the following link: https://doi.org/10.57760/sciencedb.16450.
