# Supplementary material for: Validating the Chinese version of the Apathy Motivation Index and network analysis of apathy subtypes in a healthy Chinese sample
Source: Behav Res Methods. 2025 May 12;57(6):168. doi: 10.3758/s13428-025-02686-3 (PMC12069496; doi:10.3758/s13428-025-02686-3)
Supplement: Supplementary file 1 — Supplementary file1 (DOCX 757 KB) [file 13428_2025_2686_MOESM1_ESM.docx]

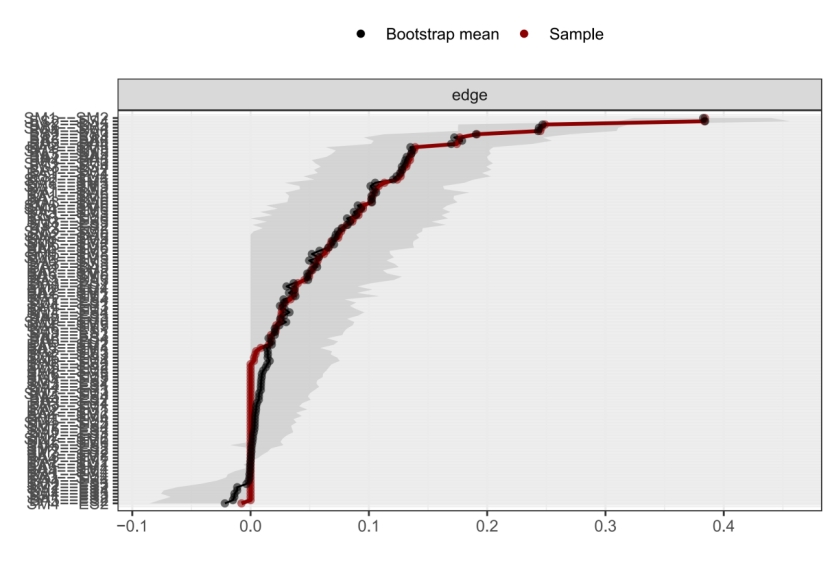


**Supplementary figure 1.**

Bootstrap test for edge weight accuracy [95% confidence interval] of apathy networks with behavioral activation, social motivation, and emotional sensitivity (n=758).


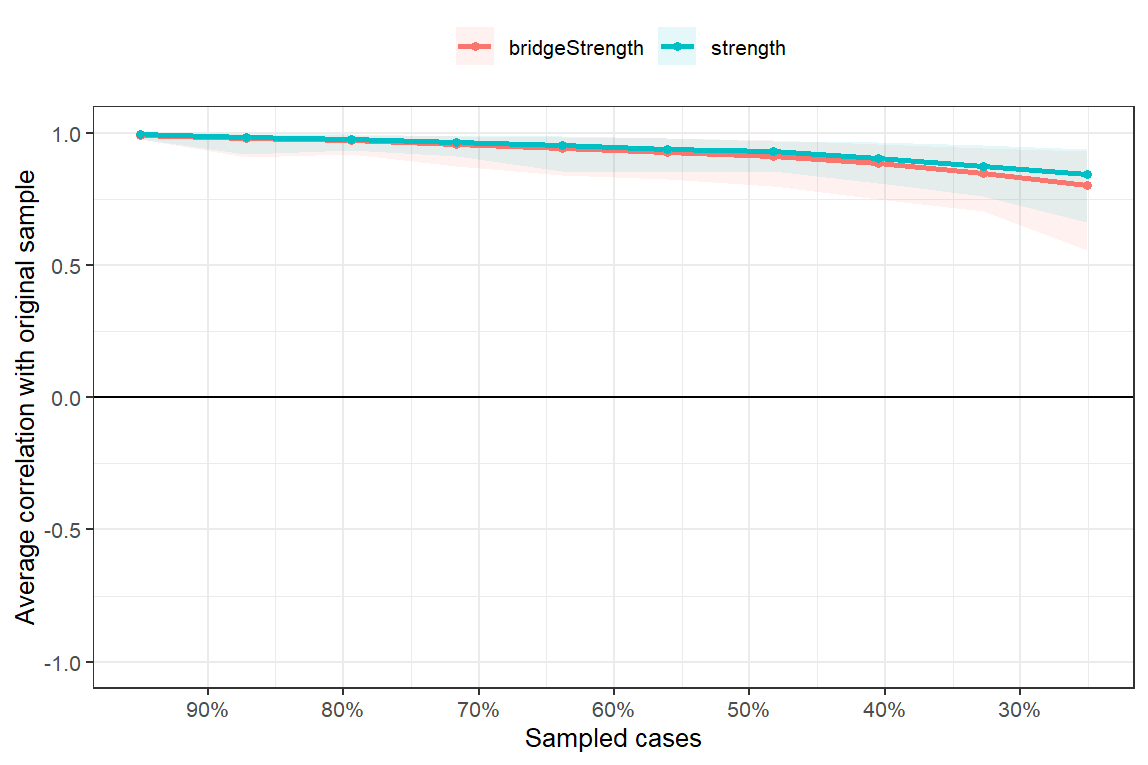
 **Supplementary Figure 2**

The stability test of the network s CS coefficient of 0.749 for strength and a CS coefficient of 0.673 for bridge strength

**English version of Apathy-Motivation Index**

| 1 | ES | I feel sad or upset when I hear bad news. |
| --- | --- | --- |
| 2 | SM | I start conversations with random people. |
| 3 | SM | I enjoy doing things with people I have just met. |
| 4 | SM | I suggest activities for me and my friends to do. |
| 5 | BA | I make decisions ﬁrmly and without hesitation. |
| 6 | ES | After making a decision, I will wonder if I have made the wrong choice. |
| 7 | ES | Based on the last two weeks, I would say I care deeply about how my loved ones think of me. |
| 8 | SM | I go out with friends on a weekly basis. |
| 9 | BA | When I decide to do something, I am able to make an effort easily. |
| 10 | BA | I don’t like to laze around. |
| 11 | BA | I get things done when they need to be done, without requiring reminders from others. |
| 12 | BA | When I decide to do something, I am motivated to see it through to the end. |
| 13 | ES | I feel awful if I say something insensitive. |
| 14 | SM | I start conversations without being prompted. |
| 15 | BA | When I have something I need to do, I do it straightaway so it is out of the way. |
| 16 | ES | I feel bad when I hear an acquaintance has an accident or illness. |
| 17 | SM | I enjoy choosing what to do from a range of activities. |
| 18 | ES | If I realise I have been unpleasant to someone, I will feel terribly guilty afterwards. |

Note: BA = Behavioural Activation. SM = Social Motivation. ES = Emotional Sensitivity.

**Chinese Version of Apathy-Motivation Index (Translated Version)**

**
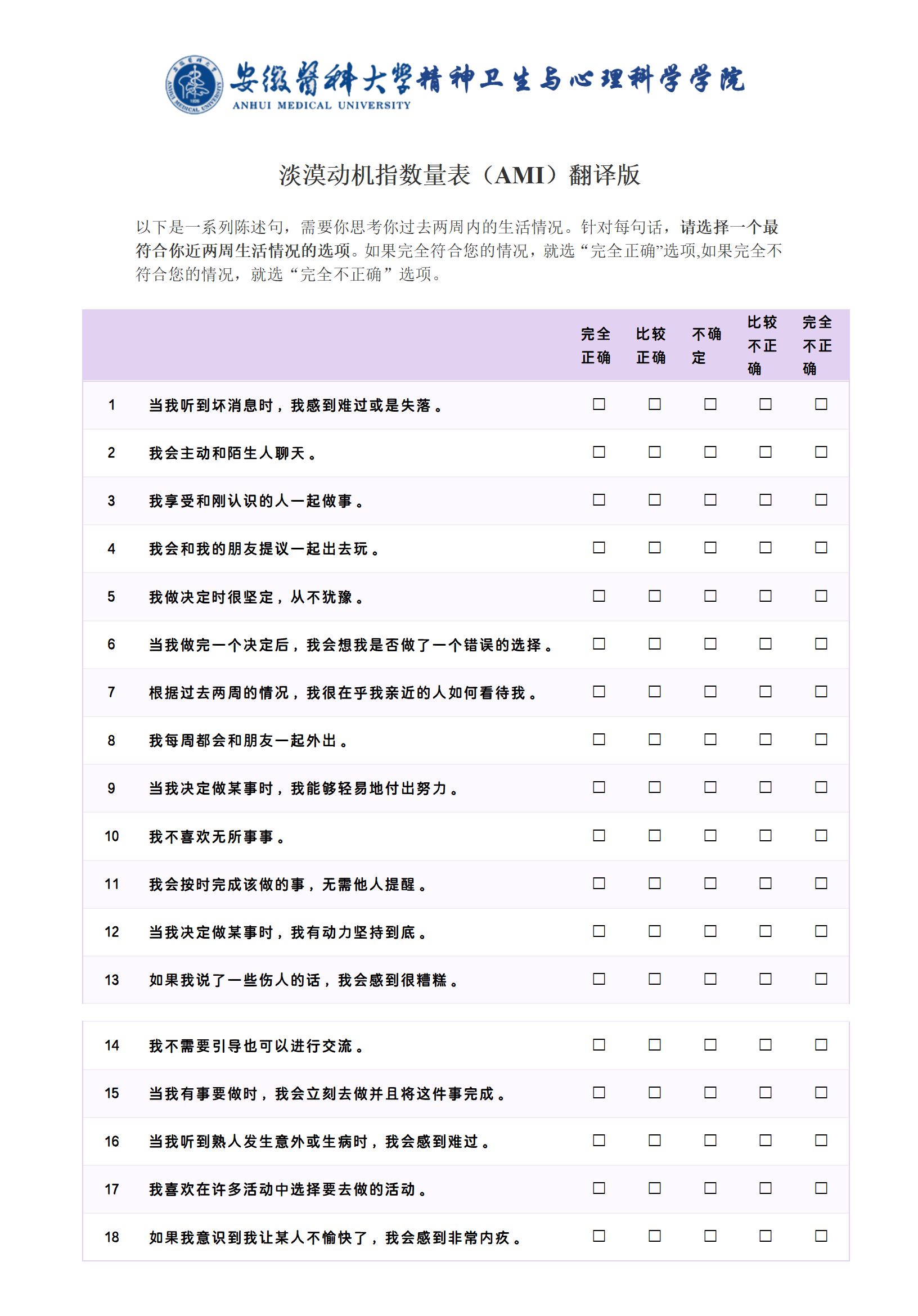
**

**Chinese Version of Apathy-Motivation Index (Revised Version)**

**
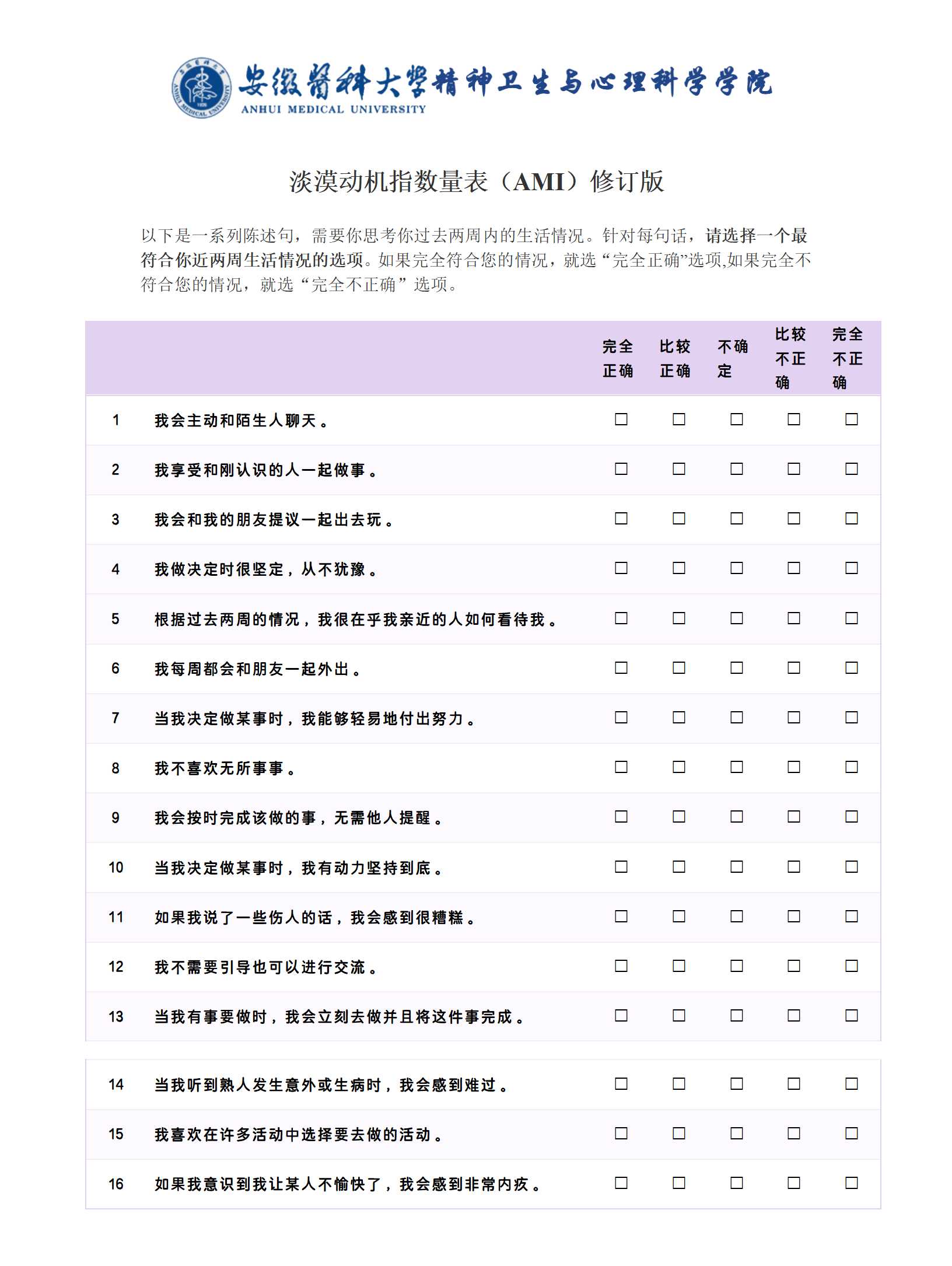
**
